# Dysglycemia Shapes Visceral Adipose Tissue’s Response to GIP, GLP-1 and Glucagon in Individuals with Obesity

**DOI:** 10.3390/metabo13050587

**Published:** 2023-04-24

**Authors:** Tiago Morais, Alexandre L. Seabra, Bárbara G. Patrício, David F. Carrageta, Marta Guimarães, Mário Nora, Pedro F. Oliveira, Marco G. Alves, Mariana P. Monteiro

**Affiliations:** 1 Endocrine and Metabolic Research, Unit for Multidisciplinary Research in Biomedicine (UMIB), University of Porto, 4050-313 Porto, Portugal; 2 Laboratory for Integrative and Translational Research in Population Health (ITR), University of Porto, 4050-313 Porto, Portugal; 3 Laboratory of Physiology, Department of Imuno-Physiology and Pharmacology, ICBAS—School of Medicine and Biomedical Sciences, University of Porto, 4050-313 Porto, Portugal; 4 Department of General Surgery, Centro Hospitalar de Entre o Douro e Vouga, 4520-220 Santa Maria da Feira, Portugal; 5 QOPNA & LAQV, Department of Chemistry, University of Aveiro, 3810-193 Aveiro, Portugal

**Keywords:** adipose tissue, metabolomics, obesity, insulin resistance, GlP-1, GIP, glucagon

## Abstract

Visceral adipose tissue (VAT) metabolic fingerprints differ according to body mass index (BMI) and glycemic status. Glucagon-like peptide 1 (GLP-1), glucose-dependent insulinotropic polypeptide (GIP) and glucagon are gut-associated hormones that play an important role in regulating energy and glucose homeostasis, although their metabolic actions in VAT are still poorly characterized. Our aim was to assess whether GLP-1, GIP and glucagon influence the VAT metabolite profile. To achieve this goal, VAT harvested during elective surgical procedures from individuals (*N* = 19) with different BMIs and glycemic statuses was stimulated with GLP-1, GIP or glucagon, and culture media was analyzed using proton nuclear magnetic resonance. In the VAT of individuals with obesity and prediabetes, GLP-1 shifted its metabolic profile by increasing alanine and lactate production while also decreasing isoleucine consumption, whereas GIP and glucagon decreased lactate and alanine production and increased pyruvate consumption. In summary, GLP-1, GIP and glucagon were shown to distinctively modulate the VAT metabolic profile depending on the subject’s BMI and glycemic status. In VAT from patients with obesity and prediabetes, these hormones induced metabolic shifts toward gluconeogenesis suppression and oxidative phosphorylation enhancement, suggesting an overall improvement in AT mitochondrial function.

## 1. Introduction

Obesity and dysglycemic disorders are both characterized by abnormal metabolic shifts that are usually exacerbated whenever the two conditions coexist. Adipose tissue (AT) is known for playing an active role in contributing to the systemic metabolic unbalances observed in affected individuals [1,2]. Indeed, visceral adipose tissue (VAT) metabolic fingerprints were previously demonstrated to differ according to the individual’s body mass index (BMI) [2,3] and glycemic status [1]. VAT glucose consumption was shown to be positively correlated with the individual’s BMI, even in the absence of insulin, whereas alanine and lactate production were negatively correlated [2]. The VAT of patients with obesity was also shown to consume less pyruvate and pyroglutamate as compared with lean counterparts, a metabolic shift that is intensified in the presence of concomitant prediabetes [1]. Additionally, higher isoleucine consumption and lower acetate production were shown to be VAT hallmarks of dysglycemia [1]. These metabolomic signatures suggest that the VAT of individuals with obesity-associated dysglycemia present an increased gluconeogenic drive and decreased mitochondrial oxidative capacity [1]. Altogether, these previous findings suggest that, while obesity leads to AT expansion and VAT metabolic reprogramming toward de novo lipogenesis [2], in the presence of dysglycemia, the glucose metabolism within the AT is also altered, being primarily characterized by mitochondrial dysfunction, which forces the cell to enhance its neoglucogenic drive [1,3].

Glucagon-like peptide 1 (GLP-1), glucose-dependent insulinotropic polypeptide (GIP) and glucagon are gut-derived peptide hormones known to be involved in energy and glucose homeostasis regulation. GLP-1 and GIP are considered incretin hormones since they were identified as acting primarily in the pancreas to stimulate glucose-dependent insulin secretion. Glucagon is a pancreatic hormone known for antagonizing insulin actions [4]. GLP-1, GIP and glucagon have been suggested to influence AT physiology, either through direct or indirect action, although the underlying mechanisms are not yet fully understood. GLP-1 primarily acts on the central nervous system to suppress food intake, thus promoting a negative energy balance, which inevitably impacts AT metabolism [5]. There is also evidence that GLP-1 has a direct effect on AT, since the GLP-1 receptor was shown to be expressed both in subcutaneous and visceral AT [6], and GLP-1 analogs were shown to induce PPAR-γ activity in a diet-dependent manner [7]. Moreover, GLP-1 was shown to promote lipolysis in 3T3-L1-derived mature adipocytes and human VAT pre-adipocytes [6]; despite the lipolytic effects observed in vitro, these findings were not replicated in vivo since subcutaneous GLP-1 infusion in healthy volunteers did not provide evidence of inducing lipolytic activity [8]. GIP effects on AT are better understood since GIP seems to be able to promote either lipogenesis [9] or lipolysis [10] depending on insulin activity [11]. In fact, GIP infusions have been shown to be able to stimulate NEFA esterification in the AT of patients with type 2 diabetes (T2D) despite being unable to stimulate insulin secretion, which occurs in euglycemic individuals [12]. Within the AT, GIP also stimulates glucose uptake [13], insulin sensitivity [14] and blood flow [15,16]. Glucagon was for a long time considered to act predominantly in the liver as a counterregulatory hormone to prevent hypoglycemia [17]. More recently, glucagon was also demonstrated to have an important role in promoting thermogenesis by acting on AT [18]. Indeed, supra-physiological concentrations of glucagon were shown to depict lipolytic activity in human AT, both in vitro [19] and in vivo [20], which seems to be abolished by insulin [21,22], while in physiological concentrations, no effect was observed whatsoever [22,23].

Given the well-known physiological roles of these gut hormones in body weight regulation, glucose homeostasis and systemic metabolism, their therapeutic potential has long been explored. As a matter of fact, these attempts have been very successful since several GLP-1 analogs have been developed and are currently widely used for the treatment of type 2 diabetes (T2D) and obesity, given their effectiveness in improving glycemic control and inducing weight loss [24]. Furthermore, the potential pharmacological utility of other gut hormones is also being tested; in particular, twincretins, or dual GLP-1/GIP analogs, are an emerging drug class that so far has been demonstrated to be superior in achieving glycemic control and body-weight loss as compared with GLP-1 analogs [25]. In addition, the potential use of co-agonist molecules with an affinity for GLP-1 and the glucagon receptor is also under investigation for obesity and T2D treatment [26,27].

Thus, the aim of this research work was to unravel the potential effects of gut peptide hormones on human AT by characterizing the effects of GLP-1, GIP and glucagon on VAT exometabolomic profiles pertaining to individuals with obesity across different glycemic statuses. The findings derived from this study could provide further insights into the potential effects of emerging gut hormone co-analogs on AT, and ultimately, this data could also contribute to understanding the role of AT in the systemic metabolic actions observed when these hormone analogs are used for obesity and T2D treatment.

## 2. Experimental Design

### 2.1. Study Subjects

Subjects undergoing elective laparoscopic interventions, gastric fundoplication, cholecystectomy or bariatric surgery for the primary treatment of hiatal hernias, cholelithiasis or obesity were invited to participate in this study. Pregnant women or patients who presented any active acute infectious or prior history of neoplastic disease were excluded. All patients enrolled signed the informed consent form before any study procedure was performed. This study was approved by the Intuitional Ethics Committee (CA 0830/16-Ot, CHEDV-HSS) and was conducted in accordance with the Local, National and European Ethical Guidelines for Medical Research involving Human Subjects.

**Figure 1:**
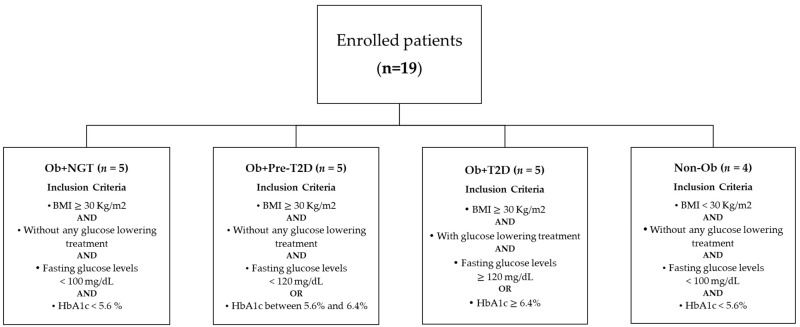
Flowchart representing individual group allocation based on patient’s anthropometric and biochemical characteristics.

Surgical procedures were performed after a minimum of 12 h of fasting. A small fragment of intra-abdominal VAT was harvested under sterile conditions during the intervention. Subjects (*N* = 19) enrolled were classified according to body corpulence depending on body mass index (BMI) and according to glycemic status depending on HbA1c levels (Figure 1). According to the BMI, subjects were either allocated into the group of subjects with obesity (*n* = 15) or without obesity (*n* = 4), while depending on fasting glucose and HbA1c levels, subjects were classified as euglycemic whenever fasting glucose levels were under 100 mg/dl, or HbA1c levels were under 5.6% in the absence of any glucose-lowering drug; as prediabetes when HbA1c levels ranged between 5.6% and 6.4% in the absence of glucose-lowering drugs; and as having type 2 diabetes (T2D) if HbA1c levels were above 6.5%. Using the above criteria, subjects were allocated into four experimental groups, namely, subjects with obesity and euglycemia (Ob + NGT, *n* = 5), subjects with obesity and pre-diabetes (Ob + Pre-T2D, *n* = 5), subjects with obesity and T2D (Ob + T2D, *n* = 5) and subjects with neither obesity nor dysglycemia who were used as controls (Non-Ob, *n* = 4). All subjects with T2D were under treatment with metformin for glycemic control until the day before surgery.

### 2.2. Adipose Tissue Isolation and Explants Incubation

After VAT harvesting, tissue fragments were rapidly processed to remove any damaged tissue debris that could be macroscopically identified, weighed and minced into smaller fragments of approximately 20 mg. VAT fragments, 1 for each tested condition, were then placed into 48-well plates in 200 µL of DMEM/F12 (12-719F, Lonza, Basel, Switzerland) and left to acclimatize in a cell culture chamber at 37 °C in the presence of 5% CO_2_ for 1 h. Afterward, culture media were discarded and replaced by fresh media, and the tissue was allowed to acclimatize for the same time period. Subsequently, VAT culture media were again replaced by fresh culture media supplemented with insulin (100 nM; Actrapid, Novo Nordisk, Bagsværd, Denmark); 1% penicillin–streptomycin (P4333, Sigma-Aldrich, St. Louis, MO, USA); and either 1, 10 or 100 nM of GLP-1 (4030663, Bachem, Bubendorf, Switzerland), GIP (4030658, Bachem, Bubendorf, Switzerland) or glucagon (4033017, Bachem, Bubendorf, Switzerland), or they were left blank without any additional hormones besides insulin. The VAT explants’ culture media were collected 48 h later and stored at −20 °C for later analysis.

### 2.3. Proton Nuclear Magnetic Resonance (^1^H-NMR)

VAT culture media metabolite content was determined with 1H-NMR using 1 mM of sodium fumarate (Sigma-Aldrich, St. Louis, MO, USA) as an internal standard (singlet, 6.50 ppm), as previously described [2]. Spectra analysis enabled us to identify and quantify the following metabolites (multiplicity, chemical shift): H1-α-glucose (doublet, 5.22 ppm), pyroglutamate (doublet of doublets, 4.16 ppm), pyruvate (singlet, 2.38 ppm), acetate (singlet, 1.90 ppm), alanine (doublet, 1.44 ppm), lactate (doublet, 1.33 ppm), isoleucine (doublet, 0.99 ppm) and valine (doublet, 0.97 ppm). The relative areas of 1H-NMR resonances were quantified using peak area integration with NUTS-Pro (Acorn NMR, Livermore, CA, USA). Spectra analysis was performed in triplicate by a single operator in order to minimize random interferences. The results are expressed as nanomoles of metabolites consumed/produced per milligram of VAT.

### 2.4. Statistical Analysis

All data are presented as mean ± standard error of the mean (SEM). Outliers were identified using the ROUT method (Q = 5%). The Shapiro–Wilk normality test was used to determine the normality of the groups. Comparison between experimental conditions with normal distribution was performed by using a repeated measures ANOVA test proceeded by Fisher’s LSD test to compare the differences between stimulated and unstimulated conditions within the same experimental group. If experimental conditions did not have a normal distribution, a Friedman test proceeded by an uncorrected Dunn’s test was used. Statistical analysis was performed using GraphPad Prism Software ver. 8.0.1 (GraphPad Software Inc., San Diego, CA, USA).

## 3. Results

### 3.1. Subjects’ Anthropometric and Clinical Features

Study subjects were predominantly female (F:M, 14:5) with a mean age of 49.6 years (range, 26–66 years) (Table 1). Besides the expected differences in body mass index (BMI) and fasting plasma glucose levels across the groups, no other significant differences were identified in the study subjects’ clinical features at the time of surgery (Table 1).

### 3.2. VAT Metabolite Profile Analysis

#### 3.2.1. Effects of GLP-1 on VAT Metabolic Profile

##### VAT Glucose and Pyruvate Consumption Is Not Influenced by GLP-1

**Figure 2:**
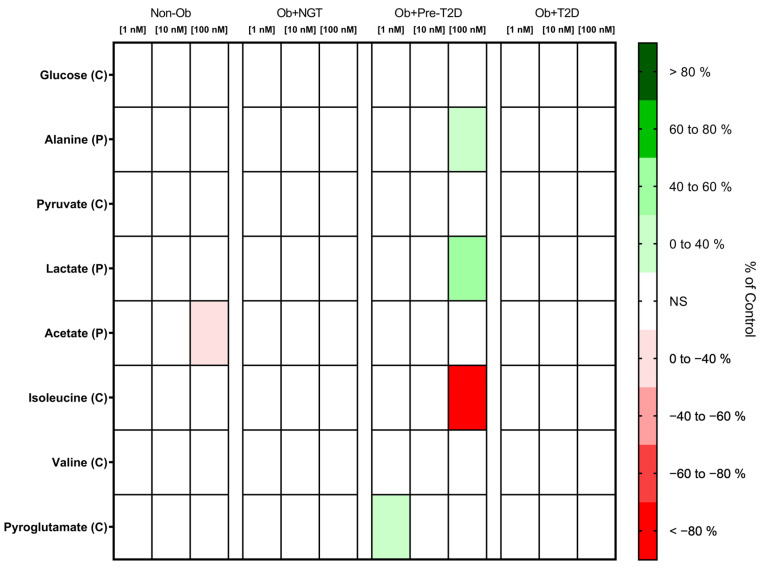
Heatmap representing the statistically significant changes (*p* < 0.05) in VAT metabolite consumption (C) and production (P) after stimulation with 1, 10 or 100 nM of GLP-1. Subjects were grouped according to body mass index (BMI) and glycemic status (with obesity and euglycemia—Ob + NGT; with obesity and prediabetes—Ob + Pre-T2D; with obesity and T2D—Ob + T2D; without obesity—Non-Ob). Results are presented as percentage changes in relation to group-specific controls (non-stimulated VAT). NS—Non-significant.

VAT glucose and pyruvate consumption were not influenced by GLP-1 stimulation in any of the study groups (Figure 2, Supplementary Material Table S1).

##### GLP-1 Decreases Acetate Production by VAT of Non-Obese Individuals, While It Increases VAT Lactate and Alanine Production of Patients with Obesity and Pre-Diabetes

The stimulation of VAT from the Non-Ob controls with GLP-1 (100 nM) decreased acetate production (Non-Ob: GLP-1 (0 nM), 2.40 ± 2.20 nmol/mg of VAT vs. GLP-1 (100 nM), 3.21 ± 2.37 nmol/mg of VAT; Δ −25.20%, *p* < 0.05), while in VAT from Ob + Pre-T2D, it increased lactate (Ob + Pre-T2D: GLP-1 (0 nM), 45.33 ± 15.61 nmol/mg of VAT vs. GLP-1 (100 nM), 65.20 ± 19.59 nmol/mg of VAT, Δ 43.83%, *p* < 0.01) and alanine production (Ob + Pre-T2D: GLP-1 (0 nM), 0.97 ± 0.40 nmol/mg of VAT vs. GLP-1 (100 nM), 1.29 ± 0.55 nmol/mg of VAT, Δ 32.56%, *p* < 0.05) (Figure 2, Table S1).

##### GLP-1 Decreases Isoleucine and Increases Pyroglutamate Consumption by VAT in Subjects with Obesity and Prediabetes

In VAT from Ob + Pre-T2D, GLP-1 (100 nM) stimulation decreased isoleucine consumption (Ob + Pre-T2D: GLP-1 (0 nM), 0.84 ± 0.06 nmol/mg of VAT vs. GLP-1 (100 nM), 0.00 ± 0.23 nmol/mg of VAT, Δ −99.45%, *p* < 0.05), and it increased pyroglutamate consumption (Ob + Pre-T2D: GLP-1 (0 nM), 12.28 ± 15.0.59 nmol/mg of VAT vs. GLP-1 (1 nM), 13.11 ± 0.81 nmol/mg of VAT, Δ 6.75%, *p* < 0.01). VAT isoleucine, pyroglutamate and valine consumption were otherwise unaltered after GLP-1 stimulation in the remaining study groups (Figure 2, Table S1).

#### 3.2.2. Effects of GIP on VAT Metabolic Profile

##### GIP Did Not Influence Glucose Consumption but Increased Pyruvate Consumption in the VAT of Subjects with Obesity and Prediabetes

**Figure 3:**
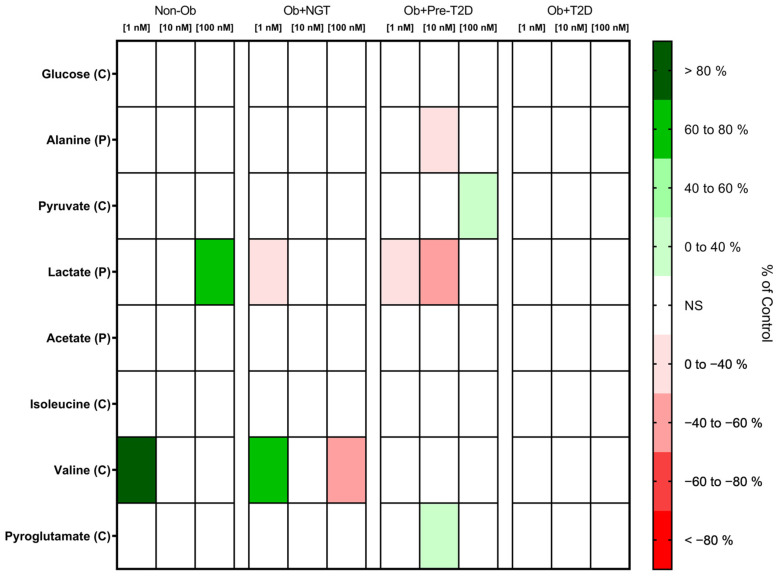
Heatmap representing the statistically significant changes (*p* < 0.05) in VAT metabolite consumption (C) and production (P) after stimulation with 1, 10 or 100 nM of GIP. Subjects were grouped according to body mass index (BMI) and glycemic status (with obesity and euglycemia—Ob + NGT; with obesity and prediabetes—Ob + Pre-T2D; with obesity and T2D—Ob + T2D; without obesity—Non-Ob). Results are presented as percentage changes in relation to group-specific controls (non-stimulated VAT). NS—Non-significant.

VAT glucose consumption remained unaltered after GIP stimulation across all experimental groups. In the VAT of the Ob + Pre-T2D group, GIP (100 nM) stimulation increased pyruvate consumption (Ob + Pre-T2D: GIP (0 nM), 2.20 ± 0.04 nmol/mg of VAT vs. GIP (100 nM), 2.46 ± 0.03 nmol/mg of VAT, Δ 11.69%, *p* < 0.01). No significant changes in VAT pyruvate consumption were observed for the other groups (Figure 3, Table S2).

##### GIP Increases Lactate Production in the VAT of Non-Obese Individuals, Whereas It Decreases Lactate and Alanine Production in the VAT of Subjects with Obesity and Prediabetes

In the VAT of the Non-Ob controls, GIP (100 nM) stimulation increased lactate production (Non-Ob: GIP (0 nM), 46.08 ± 28.48 nmol/mg of VAT vs. GIP (100 nM), 78.52 ± 36.83 nmol/mg of VAT, Δ 70.40%, *p* < 0.05). In contrast, in the VAT of the Ob + NGT and Ob + Pre-T2D groups, GIP (1 nM) stimulation decreased lactate production (Ob + NGT: GIP (0 nM), 69.39 ± 24.65 nmol/mg of VAT vs. GIP (1 nM), 45.49 ± 20.43 nmol/mg of VAT, Δ −34.44%, *p* < 0.05 and Ob + Pre-T2D: GIP (0 nM), 45.33 ± 15.61 nmol/mg of VAT vs. GIP (1 nM), 29.56 ± 13.90 nmol/mg of VAT, Δ −41.41%, *p* < 0.05). In Ob + Pre-T2D, GIP (10 nM) also decreased lactate production (Ob + Pre-T2D: GIP (0 nM), 45.33 ± 15.61 nmol/mg of VAT vs. GIP (10 nM), 25.21 ± 5.99 nmol/mg of VAT, Δ −44.39%, *p* < 0.05).

Furthermore, in the VAT of the Ob + Pre-T2D group, GIP (10 nM) decreased VAT alanine production (Ob + Pre-T2D: GIP (0 nM), 0.97 ± 0.40 nmol/mg of VAT vs. GIP (10 nM), 0.66 ± 0.31 nmol/mg of VAT, Δ −31.95%, *p* < 0.05). No significant effects on acetate production were observed after VAT stimulation with GIP in any of the study groups (Figure 3, Table S2).

##### GIP Decreases Valine Consumption in the VAT of Non-Obese Controls, and It Increases Valine and Pyroglutamate Consumption in the VAT of Patients with Obesity and Obesity with Concomitant Prediabetes, Respectively

In the VAT of Non-Ob controls, GIP (1 nM) stimulation increased valine consumption (Non-Ob: GIP (0 nM), −0.26 ± 0.38 nmol/mg of VAT vs. GIP (1 nM), 0.09 ± 0.35 nmol/mg of VAT, Δ 133.20%, *p* < 0.05). In contrast, in the VAT of subjects with Ob + NGT, a low GIP concentration (1 nM) increased valine consumption (Ob + NGT: GIP (0 nM), −0.47 ± 0.32 nmol/mg of VAT vs. GIP (1 nM), −0.18 ± 0.24 nmol/mg of VAT, Δ −60.57%, *p* < 0.05), while a high GIP concentration (100 nM) decreased valine consumption (Ob + NGT: GIP (0 nM), −0.47 ± 0.32 nmol/mg of VAT vs. GIP (100 nM), −0.69 ± 0.35 nmol/mg of VAT, Δ −47.03%, *p* < 0.05). Additionally, in the VAT of the Ob + Pre-T2D group, GIP (10 nM) stimulation increased VAT pyroglutamate consumption (Ob + Pre-T2D: GIP (0 nM), 12.28 ± 0.59 nmol/mg of VAT vs. GIP (10 nM), 13.71 ± 0.84 nmol/mg of VAT, Δ 11.67%, *p* < 0.05) (Figure 3, Table S2).

#### 3.2.3. Effects of Glucagon on VAT Metabolic Profile

##### Glucagon Increases VAT Pyruvate Consumption

**Figure 4:**
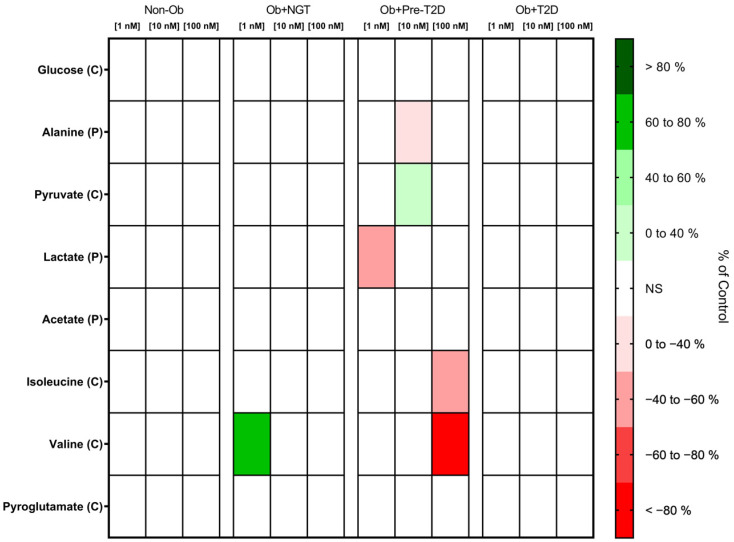
Heatmap representing the statistically significant changes (*p* < 0.05) in VAT metabolite consumption (C) and production (P) after stimulation with 1, 10 or 100 nM of glucagon. Subjects were grouped according to body mass index (BMI) and glycemic status (with obesity and euglycemia—Ob + NGT; with obesity and prediabetes—Ob + Pre-T2D; with obesity and T2D—Ob + T2D; without obesity—Non-Ob). Results are presented as percentage changes in relation to group-specific controls (non-stimulated VAT). NS—Non-significant.

Glucagon stimulation produced no significant changes in VAT glucose or pyruvate consumption, with the sole exception of the Ob + Pre-T2D group, in which glucagon stimulation (10 nM) increased VAT pyruvate consumption (Ob + Pre-T2D: glucagon (0 nM), 2.20 ± 0.04 nmol/mg of VAT vs. glucagon (10 nM), 2.44 ± 0.11 nmol/mg of VAT, Δ 10.91%, *p* < 0.05) (Figure 4, Table S3).

##### Glucagon Decreases the Lactate Production of the VAT of Subjects with Obesity and Prediabetes

In the VAT of the Ob + Pre-T2D group, glucagon (1nM) stimulation decreased lactate production (Ob + Pre-T2D: glucagon (0 nM), 45.33 ± 15.61 nmol/mg of VAT vs. glucagon (1 nM), 20.69 ± 6.06 nmol/mg of VAT, Δ −54.36%, *p* < 0.05), whereas, in a higher concentration (10 nM), it also resulted in a decrease in alanine production (Ob + Pre-T2D: glucagon (0 nM), 0.97 ± 0.40 nmol/mg of VAT vs. glucagon (10 nM), 0.70 ± 0.32 nmol/mg of VAT, Δ −28.04%, *p* < 0.05). No significant changes in lactate, alanine or acetate production were observed after glucagon stimulation in either study group (Figure 4, Table S3).

##### Glucagon Increases Valine Consumption by the VAT of Patients with Obesity, and It Also Increases Isoleucine Consumption by the VAT of Patients with Obesity and Prediabetes

In the VAT of the Ob + NGT group, after glucagon (1 nM) stimulation, valine consumption increased (Ob + NGT: glucagon (0 nM), −0.47 ± 0.32 nmol/mg of VAT vs. glucagon (1 nM), −0.12 ± 0.21 nmol/mg of VAT, Δ 73.68%, *p* < 0.05), while in the VAT of the Ob + Pre-T2D group, glucagon (100 nM) also decreased VAT isoleucine (Ob + Pre-T2D: glucagon (0 nM), 0.84 ± 0.06 nmol/mg of VAT vs. glucagon (100 nM), 0.37 ± 0.30 nmol/mg of VAT, Δ −55.17%, *p* < 0.05) and valine consumption (Ob + Pre-T2D: Glucagon (0 nM), 0.15 ± 0.43 nmol/mg of VAT vs. Glucagon (100 nM), −0.10 ± 0.42 nmol/mg of VAT, Δ −169.2%, *p* < 0.01). Glucagon stimulation produced no significant changes in valine, isoleucine or pyroglutamate consumption by the VAT of the other studied groups (Figure 4, Table S3).

## 4. Discussion

Obesity is characterized by an abnormal AT expansion, alongside the severe impairment of AT physiological functions [28]. In particular, VAT expansion and dysfunction are associated with several systemic metabolic unbalances, mostly driven by or related to insulin resistance [29]. Insulin plays a major role in modulating glucose uptake/consumption by peripheral tissues, such as skeletal muscle and AT. Furthermore, insulin is also able to regulate the metabolic fate of glucose by promoting gluconeogenesis or de novo lipogenesis while suppressing catabolic processes, such as lipolysis [30]. In AT, when insulin signaling is impaired, fatty acid uptake and synthesis are decreased, while the rate of lipolysis increases [31]. This metabolic pattern with free fatty acid overload hampers mitochondrial health, further impairing AT functions [31]. We previously demonstrated that the VAT metabolic profile of patients with obesity and prediabetes presented metabolic shifts, suggesting an enhanced gluconeogenic drive, likely due to compromised mitochondrial oxidative capacity [1,3].

GLP-1 analogs were the first gut hormone-based drugs to become available for T2D and obesity treatment. Since then, several other molecules that, along with GLP-1, also target GIP or glucagon receptors have been developed and are currently being tested in clinical trials for the treatment of obesity and other obesity-related disorders, such as T2D [32]. The weight loss and glycemic effectiveness attributed to these novel dual agonist molecules have shown they are able to largely surpass the outcomes of previous trials with isolated GLP-1 agonists [33,34]. Therefore, there is great interest in understanding the mechanisms underlying the weight loss and metabolic effects observed in clinical trials by exploring how other organs, besides the pancreas and brain, are targeted by these molecules in achieving these systemic metabolic effects. In particular, there is an unmet need to further understand how AT is influenced by native gut hormones, namely, GLP-1, GIP and glucagon. For this, we conducted an in vitro study in which the VAT of individuals with obesity with different glycemic statuses and normal-weight euglycemic control was exposed to GLP-1, GIP and glucagon in different concentrations. It should be noticed that the high concentrations of hormones that were used in this study were intended to replicate pharmacological doses and not mere physiological conditions.

In the VAT of normal-weight euglycemic individuals, high concentrations of GLP-1 were able to decrease acetate production, whereas high concentrations of GIP were able to increase lactate production. Furthermore, low concentrations of GIP were also able to increase valine consumption, suggesting that GIP acts synergistically with insulin on AT to suppress lipolysis [35]. Neither of the metabolic effects induced by GLP-1 or GIP was observed in the VAT of subjects with obesity with or without concomitant dysglycemia.

**Figure 5:**
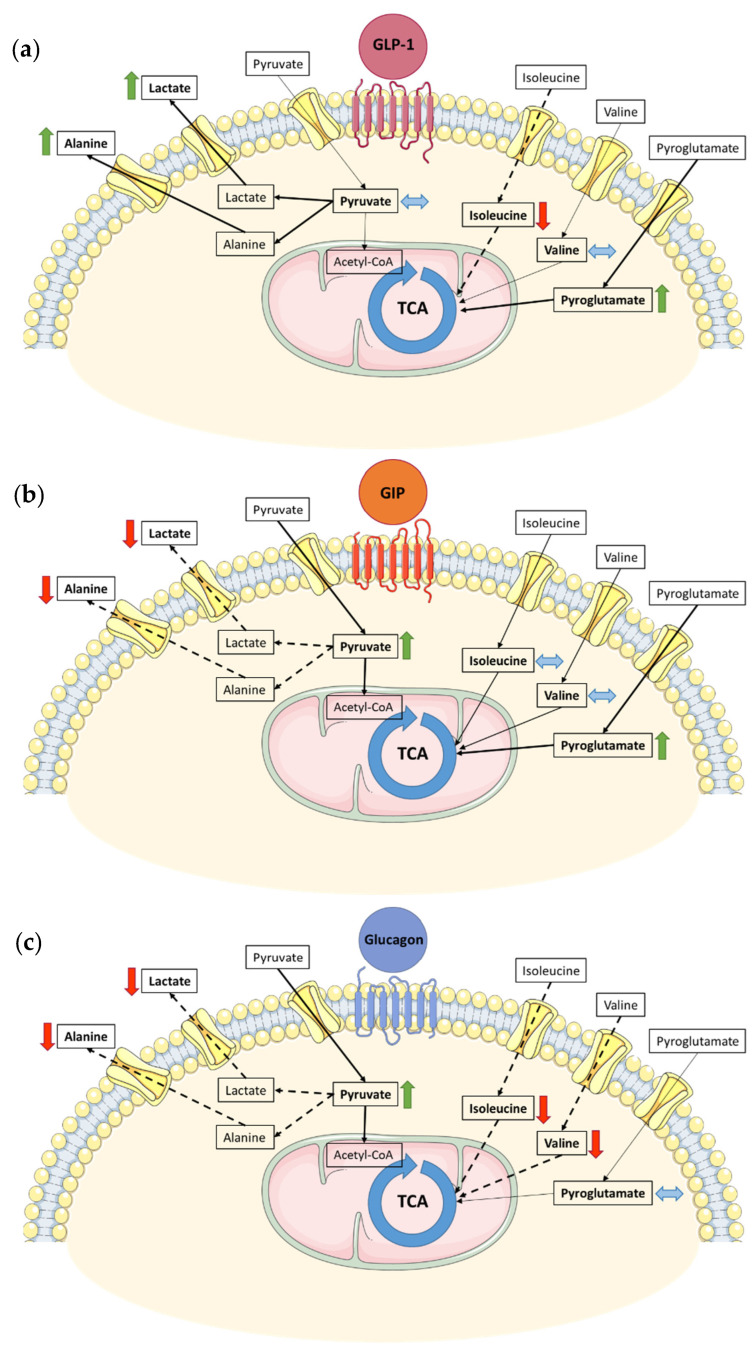
Putative effects of GLP-1 (**a**), GIP (**b**) and glucagon (**c**) stimulation on the VAT metabolism of subjects with obesity and prediabetes. GLP-1 stimulation leads to an increase in alanine/lactate production while decreasing isoleucine consumption, suggesting the suppression of gluconeogenesis and fatty acid production (**a**). GIP stimulation decreases both lactate and alanine production, coupled with an increase in pyruvate consumption. This metabolic pattern suggests a shift toward oxidative phosphorylation or de novo lipogenesis (**b**). Glucagon increased VAT pyruvate consumption, along with a decrease in alanine/lactate production and reduced branched-chain amino acid consumption (**c**). TCA—tricarboxylic acid cycle; Acetyl-CoA—acetyl coenzyme A.

In the VAT of individuals with obesity and prediabetes, GLP-1 shifted the VAT metabolic profile by increasing lactate and alanine production while decreasing isoleucine consumption. Moreover, GLP-1 increased pyroglutamate consumption. Lactate assumes an important role in whole-body metabolism since it can replace glucose as an energy source [36]. Therefore, an increase in lactate and alanine production suggests a possible metabolic shift toward anaerobic glycolysis, which, in turn, diverts metabolism from acetyl-CoA production and oxidative phosphorylation [37]. Additionally, lactate can act synergistically with insulin in order to suppress lipolysis within the AT [35]. Decreasing lipolysis further improves AT mitochondrial health since it limits beta-oxidation and oxidative phosphorylation, thus reducing oxidative stress [38]. Furthermore, GLP-1 decreases isoleucine consumption. This has the potential to further limit substrate availability to fuel the tricarboxylic acid cycle (TCA), thus further decreasing oxidative phosphorylation [39]. Therefore, the metabolic shifts induced by GLP-1 suggest that there could be a suppression of gluconeogenesis and mitochondrial activity, which would ultimately decrease the metabolic burden in a putative attempt to protect AT mitochondrial health, although this hypothesis would need to be confirmed by further studies (Figure 5a).

In contrast, GIP effects on the VAT of individuals with obesity and prediabetes seem to mirror the effects of GLP-1 by decreasing lactate and alanine production while increasing pyruvate and pyroglutamate consumption. These effects were largely replicated by glucagon, which also decreased lactate and alanine production, coupled with increased pyruvate consumption. GIP-induced metabolic shifts point toward an increase in acetyl-CoA production, which will feed the TCA and eventually promote de novo lipogenesis, thus increasing the AT’s lipid-storing capability [40] in a way that seems to mimic the effect of insulin in AT bioenergetics [31]. It is worth noting that GIP also increased pyroglutamate consumption—used here as a proxy for glutamine levels [41]—thus increasing the capacity to limit oxidative damage and offsetting the possible increase in mitochondrial activity [42] (Figure 5b). Glucagon’s metabolic effects on AT, besides mimicking GIP effects, were also shown to be dose-dependent. Indeed, in lower doses, glucagon decreased lactate and alanine production and increased pyruvate consumption, while at higher doses, glucagon also decreased the consumption of branched-chain amino acids (BCAAs), valine and isoleucine.

Glucagon has been long recognized for stimulating AT lipolysis [19] by suppressing insulin action [19,43]. However, since a high insulin concentration was present in the culture media, this could have been enough to counteract the lipogenic activity of glucagon even though the insulin signaling was likely to be impaired, thus providing an explanation for the fact that no metabolic shifts were observed in that direction [19]. Nonetheless, glucagon has also been demonstrated to stimulate thermogenic activity [44], both through brown AT-dependent [45] and -independent mechanisms [46], whereas white adipocyte enters a re-esterification/lipolysis cycle in order to provide fuel and generate heat [47]. In our experimental model, glucagon increased pyruvate consumption, suggesting that this substrate could be used as fuel to dissipate energy. In contrast, in higher concentrations, glucagon metabolic effects shifted toward BCAA trafficking by decreasing its consumption. The decreased consumption of BCAA could derive from a decreased gluconeogenesis drive, with substrate diversion toward the mitochondria to feed the TCA cycle as a means of dissipating energy as part of thermogenic processes [48]. Overall, GIP and glucagon induced the VAT metabolite profile, suggesting metabolic shifts that culminate in oxidative phosphorylation (Figure 5c).

In sum, these data suggest that, in individuals with obesity and insulin resistance, GLP-1, GIP and glucagon are likely to increase AT’s metabolic efficiency despite this effect being attained via different mechanisms and potentially acting in synergy. Of note, despite metabolic profile modifications—suggesting improved mitochondrial function known to be hampered in patients with obesity and prediabetes—in the tested conditions, none of the hormones were able to fully revert the VAT metabolic phenotype to the euglycemic profile.

Interestingly enough, in patients with obesity and T2D, none of the studied hormones produced any significant metabolic profile modifications. Moreover, the metabolic profile of the VAT of patients with obesity and T2D was almost identical to the one detected in the VAT from individuals without obesity and euglycemia, replicating our previous findings [1]. We hypothesize that this could have been attributed to the fact that patients were under metformin treatment since the disease was diagnosed [49], which could have enabled VAT to restore its metabolic health.

This work also presented some experimental patient-related and methodological challenges that can limit the interpretation of the data. The first limitation is the small number of subjects per experimental group, which we tried as much as possible to mitigate by pairing patients according to clinical features in order to avoid confounding factors such as age, gender and anthropometric parameters and using an appropriate statistical methodology; nevertheless, this limitation could still have led to some data skewing. In addition, the Non-Ob group is not an ideal control since subjects were overweight and had elevated triglyceride levels, reflecting the high prevalence of these conditions in the general population. That notwithstanding, it must be acknowledged that both conditions reflect a metabolic dysfunctional state and thus are potentially already associated with VAT metabolic shifts. Therefore, this fact could have influenced the results, including masking metabolite differences that could have been identified if metabolically healthy, normal-weight individuals were used as controls, for instance, as observed for glucagon. Nonetheless, our study proved to be sufficiently robust in identifying intragroup shifts in VAT metabolite profiles induced by gut hormones. Despite these limitations, patients presented clearly different phenotypic profiles with no overlaps when compared with the other groups. Another limitation was the fact that we had to rely on pre-operative biochemical and clinical evaluations to determine the patients’ metabolic statuses, as these parameters were not assessed on the same day that surgery took place. Still, we believe these parameters are sufficiently reliable to categorize individuals into the different experimental groups. The methodological limitations are related to the fact that we used ^1^H-NMR to evaluate the metabolic profile, which, although it allowed us to assess small metabolites that would go undetected using other methods, is not as powerful as other techniques, such as mass spectrometry [50]. Moreover, we decided to use whole-tissue VAT to evaluate metabolite dynamics regardless of the cell type responsible for the effects observed in order to mimic ex vivo as much as possible in vivo conditions, as well as supra-physiological concentrations of insulin and gut hormones in order to increase the chances of reaching the inner tissue and not just cells at the periphery. However, the fact that we created a hyperinsulinemic environment, which is a powerful regulator of adipose tissue metabolism, could have overwhelmed the cellular response and potentially masked the AT response to the other gut hormones. Another aspect that could have influenced our results was the 48 h incubation time of our VAT explants. Although the tissue was macroscopically and microscopically sound after 48 h, the incubation times could produce overstimulation in the tissue in response not only to GLP-1/GIP/glucagon but also insulin. Reassuring is the fact that previous studies also used a 48h stimulation with insulin or GLP-1/GIP without deleterious effects observed [51,52,53]. Despite these limitations, the study also has the strength of having simulated insulin-resistance conditions with high glucose and insulin levels, as well as testing the effect of gut hormones in concentrations that are comparable to pharmacological doses [54], which provided novel insights into the crosstalk between insulin signaling and gut hormone action in VAT.

More than providing answers, this study raises several questions that need to be addressed in the future. Here, we assessed the metabolites that are present at extracellular media after GLP-1, GIP or glucagon stimulation, which only act as surrogates of intracellular metabolic shifts and ought to be explored. Furthermore, this study’s results point toward changes in mitochondrial functions, which need to be directly assessed using techniques such as those involving a Seahorse analyzer. Indeed, our group has already explored how BMI influences mitochondrial function in the VAT of individuals with obesity [3]. Additionally, in vivo studies will be needed to support our newly raised hypothesis and confirm these findings. Finally—and perhaps most relevant in the advent of the forthcoming gut hormone dual agonist drugs for T2D and obesity treatment—there is the unmet need to address how hormone combinations modify VAT metabolic fingerprints and to what extent these contribute or are clinically relevant to the weight loss and systemic metabolic effects observed.

While the VAT metabolic shifts induced by GLP-1, GIP and glucagon can be difficult to translate into a clinical perspective, data suggest that, although they act through different mechanisms, GLP-1, GIP and glucagon may contribute to restoring AT physiology in patients with obesity and insulin resistance. There is still the need to further examine the effects of hormone combinations on VAT’s mitochondrial functions, as well as on glucose and lipid metabolism.

## 5. Conclusions

In summary, although we were not able to fully revert the altered phenotype, GLP-1, GIP and glucagon did improve the metabolic profile of the VAT of subjects with obesity and prediabetes. These findings provide additional evidence in support of the effect of these hormones on AT and a rationale for the potential mechanisms by which GLP-1/GIP and GLP-1/glucagon dual agonist drugs can achieve metabolic benefits in patients with obesity and T2D.

## Figures and Tables

**Table 1:** Study subjects’ anthropometric, clinical and biochemical features.

	Ob + NGT (*n* = 5)	Ob + Pre-T2D (*n* = 5)	Ob + T2D (*n* = 5)	Non-Ob (*n* = 4)
Age (years)	44 ± 7	50 ± 3	56 ± 2	48 ± 7
Sex (F%)	80%	80%	80%	50%
BMI (kg/m^2^)	41.4 ± 2.6 ***	44.0 ± 2.8 ***	41.5 ± 2.5 ***	26.1 ± 1.0
Fasting glucose (mg/dL)	93.8 ± 0.8	109.2 ± 8.0	161.4 ± 26.8 *^,†^	88.8 ± 3.2
HbA1c (%)	5.4 ± 0.2	6.2 ± 0.2	7.6 ± 1.3 ^†^	n.a.
Metformin (%)	0%	0%	100%	0%
SBP (mmHg)	137 ± 7	150 ± 6	146 ± 4	133 ± 7
DBP (mmHg)	79 ± 4	85 ± 3	73 ± 4	80 ± 4
Total cholesterol (mg/dL)	161 ± 7	220 ± 15	206 ± 24	177 ± 25
HDL (mg/dL)	52 ± 5	47 ± 7	48 ± 5	35 ± 11
LDL (mg/dL)	96.4 ± 10.3	142.4 ± 7.2	135.4 ± 23.2	100.9 ± 6.7
Triglycerides (mg/dL)	102 ± 8	111 ± 22	203 ± 49	206 ± 147

Subjects were grouped according to body mass index (BMI) and glycemic status (with obesity and euglycemia—Ob + NGT; with obesity and prediabetes—Ob + Pre-T2D; with obesity and T2D—Ob + T2D; without obesity—Non-Ob). HbA1c—hemoglobin A1c; SBP—systolic blood pressure; DBP—diastolic blood pressure; HDL—high-density lipoprotein; LDL—low-density lipoprotein; n.a.—Not available. Data are presented as mean ± SEM. * vs. Non-Ob (*, *p* < 0.05; ***, *p* < 0.001); ^†^ vs. Ob + NGT (^†^, *p* < 0.05).

## Data Availability

Data is available in an article and supplementary material.

## Supplementary Materials

The following supporting information can be downloaded at https://www.mdpi.com/article/10.3390/metabo13050587/s1: Table S1: VAT metabolite consumption or production (nmol/mg of VAT) in response to 1, 10 or 100 nM GLP-1 stimulation; Table S2: VAT metabolite consumption or production (nmol/mg of VAT) in response to 1, 10 or 100 nM GIP stimulation; Table S3: VAT metabolite consumption or production (nmol/mg of VAT) in response to 1, 10 or 100 nM glucagon stimulation.
