# Supplementary material for: Dysglycemia Shapes Visceral Adipose Tissue’s Response to GIP, GLP-1 and Glucagon in Individuals with Obesity
Source: Metabolites. 2023 Apr 24;13(5):587. doi: 10.3390/metabo13050587 (PMC10224016; doi:10.3390/metabo13050587)
Supplement: Supplementary file 1 [file metabolites-13-00587-s001.zip › metabolites-2259430-SI.pdf]

Supplemental Material

Table S1. VAT metabolite consumption or production (nmol/mg of VAT) in response to 1, 10 or 100 nM GLP-1 stimulation.

Non-Ob (n=4)

[GLP-1]	Glucose (C)	Pyruvate (C)	Lactate (P)	Acetate (P)	Alanine (P)	Isoleucine (C)	Valine (C)	Pyroglutamate (C)
[0 nM]	22.92 ± 9.42	3.31 ± 0.16	46.08 ± 28.48	3.21 ± 2.37	0.76 ± 0.56	0.47 ± 0.48	-0.26 ± 0.38	17.18 ± 0.62
[1 nM]	37.05 ± 7.15	3.10 ± 0.31	58.28 ± 3.08	3.13 ± 3.93	1.01 ± 0.21	0.68 ± 0.32	0.11 ± 0.35	16.63 ± 2.19
[10 nM]	19.91 ± 13.93	2.34 ± 0.47	37.63 ± 34.87	2.12 ± 2.56	0.73 ± 0.20	0.34 ± 0.41	-0.13 ± 0.27	16.76 ± 3.30
[100 nM]	28.62 ± 14.01	2.95 ± 0.45	66.65 ± 32.67	2.40 ± 2.20*	0.90 ± 0.01	0.26 ± 0.26	-0.12 ± 0.40	16.11 ± 2.23

Ob+NGT (n=5)

[GLP-1]	Glucose (C)	Pyruvate (C)	Lactate (P)	Acetate (P)	Alanine (P)	Isoleucine (C)	Valine (C)	Pyroglutamate (C)
[0 nM]	29.00 ± 13.95	2.74 ± 0.30	69.39 ± 24.65	1.85 ± 3.14	1.19 ± 0.73	0.18 ± 0.39	-0.47 ± 0.32	14.17 ± 1.38
[1 nM]	37.71 ± 17.77	2.72 ± 0.36	73.04 ± 31.85	2.50 ± 3.00	1.34 ± 0.59	0.22 ± 0.32	-0.30 ± 0.38	14.42 ± 1.37
[10 nM]	32.91 ± 21.95	2.59 ± 0.36	75.87 ± 41.56	2.26 ± 3.60	1.27 ± 0.59	0.08 ± 0.43	-0.53 ± 0.26	14.38 ± 1.71
[100 nM]	29.78 ± 21.94	2.70 ± 0.35	82.20 ± 41.80	1.14 ± 0.78	1.27 ± 0.62	-0.15 ± 0.30	-0.66 ± 0.33	14.14 ± 1.45

Ob+Pre-T2D (n=5)

[GLP-1]	Glucose (C)	Pyruvate (C)	Lactate (P)	Acetate (P)	Alanine (P)	Isoleucine (C)	Valine (C)	Pyroglutamate (C)
[0 nM]	30.41 ± 8.36	2.20 ± 0.04	45.33 ± 15.61	-0.12 ± 0.55	0.97 ± 0.40	0.84 ± 0.06	0.15 ± 0.43	12.28 ± 0.59
[1 nM]	27.27 ± 5.30	2.38 ± 0.18	40.82 ± 17.64	-0.12 ± 0.77	0.94 ± 0.33	0.56 ± 0.50	0.03 ± 0.43	13.11 ± 0.81*
[10 nM]	27.12 ± 13.86	2.27 ± 0.15	46.57 ± 10.16	-0.22 ± 0.48	0.77 ± 0.32	0.43 ± 0.30	0.07 ± 0.09	13.09 ± 1.55
[100 nM]	34.32 ± 9.35	2.45 ± 0.34	65.20 ± 19.59**	0.44 ± 1.13	1.29 ± 0.55*	0.00 ± 0.23*	-0.09 ± 0.35	13.44 ± 1.80

Ob+T2D (n=5)

[GLP-1]	Glucose (C)	Pyruvate (C)	Lactate (P)	Acetate (P)	Alanine (P)	Isoleucine (C)	Valine (C)	Pyroglutamate (C)
[0 nM]	28.86 ± 14.04	2.67 ± 0.37	61.07 ± 23.80	1.68 ± 0.56	1.05 ± 0.52	0.16 ± 0.39	-0.30 ± 0.47	14.22 ± 1.52
[1 nM]	27.51 ± 7.56	2.67 ± 0.38	55.82 ± 25.24	2.24 ± 1.36	0.96 ± 0.36	0.19 ± 0.28	-0.21 ± 0.46	14.01 ± 1.41
[10 nM]	33.29 ± 16.17	2.74 ± 0.39	66.33 ± 42.55	1.40 ± 0.90	1.00 ± 0.56	0.27 ± 0.15	-0.32 ± 0.45	14.07 ± 1.74
[100 nM]	27.52 ± 12.77	2.64 ± 0.66	61.92 ± 31.35	1.48 ± 0.94	1.08 ± 0.39	0.30 ± 0.25	-0.48 ± 0.29	14.17 ± 1.59

(C)—metabolite consumption; (P)—metabolite production. Subjects were grouped according to body mass index (BMI) and glycemic status (with obesity and euglycemia—Ob+NGT; with obesity and pre-diabetes—Ob+Pre-T2D; with obesity and T2D—Ob+T2D; without obesity—Non-Ob). Data presented as mean ± SEM. * $p < 0.05$ or ** $p < 0.01$ vs GLP-1 [0 nM] of respective experimental group.

Table S2. VAT metabolite consumption or production (nmol/mg of VAT) in response to 1, 10 or 100 nM GIP stimulation.

Non-Ob (n=4)

[GIP]	Glucose (C)	Pyruvate (C)	Lactate (P)	Acetate (P)	Alanine (P)	Isoleucine (C)	Valine (C)	Pyroglutamate (C)
[0 nM]	22.92 ± 9.42	3.31 ± 0.16	46.08 ± 28.48	3.21 ± 2.37	0.76 ± 0.56	0.47 ± 0.48	-0.26 ± 0.38	17.18 ± 0.62
[1 nM]	25.45 ± 12.33	2.86 ± 0.73	39.65 ± 20.60	2.26 ± 4.09	0.79 ± 0.30	0.70 ± 0.39	0.09 ± 0.35*	17.33 ± 3.44
[10 nM]	32.67 ± 5.41	3.18 ± 0.17	47.45 ± 11.95	1.95 ± 2.79	0.76 ± 0.23	0.81 ± 0.18	0.27 ± 0.15	17.02 ± 2.06
[100 nM]	33.82 ± 18.87	2.94 ± 0.96	78.52 ± 36.83*	1.09 ± 1.27	1.25 ± 0.34	0.40 ± 0.40	-0.23 ± 0.57	17.71 ± 4.12

Ob+NGT (n=5)

[GIP]	Glucose (C)	Pyruvate (C)	Lactate (P)	Acetate (P)	Alanine (P)	Isoleucine (C)	Valine (C)	Pyroglutamate (C)
[0 nM]	29.00 ± 13.95	2.74 ± 0.3	69.39 ± 24.65	1.85 ± 3.14	1.19 ± 0.73	0.18 ± 0.39	-0.47 ± 0.32	14.17 ± 1.38
[1 nM]	26.93 ± 12.14	2.50 ± 0.23	45.49 ± 20.43*	1.49 ± 2.17	0.79 ± 0.44	0.29 ± 0.33	-0.18 ± 0.24*	14.35 ± 0.14
[10 nM]	47.82 ± 17.49	2.65 ± 0.13	92.99 ± 34.15	2.57 ± 3.06	1.42 ± 0.57	0.16 ± 0.43	-0.54 ± 0.30	13.99 ± 0.57
[100 nM]	24.16 ± 14.96	2.81 ± 0.37	65.85 ± 29.79	0.89 ± 1.39	1.28 ± 0.71	-0.07 ± 0.27	-0.69 ± 0.35*	14.93 ± 1.71

Ob+Pre-T2D (n=5)

[GIP]	Glucose (C)	Pyruvate (C)	Lactate (P)	Acetate (P)	Alanine (P)	Isoleucine (C)	Valine (C)	Pyroglutamate (C)
[0 nM]	30.41 ± 8.36	2.20 ± 0.04	45.33 ± 15.61	-0.12 ± 0.55	0.97 ± 0.40	0.84 ± 0.06	0.15 ± 0.43	12.28 ± 0.59
[1 nM]	20.45 ± 4.80	2.37 ± 0.36	29.56 ± 13.90*	-0.39 ± 0.52	0.72 ± 0.33	0.56 ± 0.47	0.06 ± 0.49	13.60 ± 1.20
[10 nM]	23.74 ± 4.49	2.36 ± 0.16	25.21 ± 5.99*	-0.59 ± 0.19	0.66 ± 0.31*	0.70 ± 0.30	0.22 ± 0.29	13.71 ± 0.84*
[100 nM]	24.93 ± 6.85	2.46 ± 0.03**	44.85 ± 7.77	-0.22 ± 0.53	0.88 ± 0.50	0.56 ± 0.21	0.06 ± 0.25	13.29 ± 1.74

Ob+T2D (n=5)

[GIP]	Glucose (C)	Pyruvate (C)	Lactate (P)	Acetate (P)	Alanine (P)	Isoleucine (C)	Valine (C)	Pyroglutamate (C)
[0 nM]	28.86 ± 14.04	2.67 ± 0.37	61.07 ± 23.80	1.68 ± 0.56	1.05 ± 0.52	0.16 ± 0.39	-0.30 ± 0.47	14.22 ± 1.52
[1 nM]	30.21 ± 12.03	2.70 ± 0.39	49.30 ± 15.88	1.89 ± 1.74	0.77 ± 0.09	0.53 ± 0.15	0.03 ± 0.19	14.85 ± 1.70
[10 nM]	28.24 ± 10.26	2.54 ± 0.54	45.42 ± 21.07	1.96 ± 1.46	0.78 ± 0.28	0.41 ± 0.22	-0.13 ± 0.08	13.93 ± 1.21
[100 nM]	25.14 ± 8.74	2.33 ± 0.18	50.62 ± 19.69	1.05 ± 0.57	0.94 ± 0.16	0.26 ± 0.10	-0.25 ± 0.17	13.56 ± 0.88

(C)—metabolite consumption; (P)—metabolite production. Subjects were grouped according to body mass index (BMI) and glycemic status (with obesity and euglycemia—Ob+NGT; with obesity and pre-diabetes—Ob+Pre-T2D; with obesity and T2D—Ob+T2D; without obesity—Non-Ob). Data presented as mean ± SEM. * $p < 0.05$ or ** $p < 0.01$ vs GLP-1 [0 nM] of respective experimental group.

Table S3. VAT metabolite consumption or production (nmol/mg of VAT) in response to 1, 10 or 100 nM glucagon stimulation.

Non-Ob (n=4)

[Glucagon]	Glucose (C)	Pyruvate (C)	Lactate (P)	Acetate (P)	Alanine (P)	Isoleucine (C)	Valine (C)	Pyroglutamate (C)
[0 nM]	22.92 ± 9.42	3.31 ± 0.16	46.08 ± 28.48	3.21 ± 2.37	0.76 ± 0.56	0.47 ± 0.48	-0.26 ± 0.38	17.18 ± 0.62
[1 nM]	22.32 ± 9.55	2.39 ± 0.58	35.60 ± 18.62	1.76 ± 2.68	0.83 ± 0.30	0.38 ± 0.39	-0.11 ± 0.31	15.78 ± 3.09
[10 nM]	27.43 ± 11.02	3.09 ± 0.43	45.41 ± 12.19	1.73 ± 2.85	0.87 ± 0.35	0.65 ± 0.44	-0.02 ± 0.53	16.23 ± 1.42
[100 nM]	28.60 ± 8.57	2.69 ± 0.24	54.28 ± 21.30	1.33 ± 0.98	1.06 ± 0.09	0.29 ± 0.20	-0.13 ± 0.30	17.38 ± 3.37

Ob+NGT (n=5)

[Glucagon]	Glucose (C)	Pyruvate (C)	Lactate (P)	Acetate (P)	Alanine (P)	Isoleucine (C)	Valine (C)	Pyroglutamate (C)
[0 nM]	29.00 ± 13.95	2.74 ± 0.30	69.39 ± 24.65	1.85 ± 3.14	1.19 ± 0.73	0.18 ± 0.39	-0.47 ± 0.32	14.17 ± 1.38
[1 nM]	33.61 ± 12.75	2.78 ± 0.30	58.17 ± 26.88	2.42 ± 3.97	1.07 ± 0.73	0.37 ± 0.24	-0.12 ± 0.21*	15.35 ± 1.19
[10 nM]	22.62 ± 9.57	2.25 ± 0.81	51.65 ± 16.92	-0.38 ± 0.14	0.82 ± 0.66	0.32 ± 0.24	-0.22 ± 0.36	14.47 ± 1.10
[100 nM]	36.11 ± 17.37	2.82 ± 0.32	93.60 ± 32.38	0.47 ± 1.07	1.50 ± 0.76	-0.21 ± 0.12	-0.81 ± 0.22	14.79 ± 1.18

Ob+Pre-T2D (n=5)

[Glucagon]	Glucose (C)	Pyruvate (C)	Lactate (P)	Acetate (P)	Alanine (P)	Isoleucine (C)	Valine (C)	Pyroglutamate (C)
[0 nM]	30.41 ± 8.36	2.20 ± 0.04	45.33 ± 15.61	-0.12 ± 0.55	0.97 ± 0.40	0.84 ± 0.06	0.15 ± 0.43	12.28 ± 0.59
[1 nM]	23.86 ± 13.11	2.37 ± 0.52	20.69 ± 6.06*	0.33 ± 1.04	0.78 ± 0.20	0.54 ± 0.33	0.06 ± 0.29	14.19 ± 1.80
[10 nM]	30.22 ± 9.23	2.44 ± 0.11*	36.79 ± 19.38	-0.60 ± 0.18	0.70 ± 0.32*	0.82 ± 0.24	0.24 ± 0.27	13.49 ± 0.99
[100 nM]	36.35 ± 12.42	2.44 ± 0.33	49.48 ± 8.93	0.47 ± 1.06	1.12 ± 0.41	0.37 ± 0.30*	-0.10 ± 0.42**	13.42 ± 1.28

Ob+T2D (n=5)

[Glucagon]	Glucose (C)	Pyruvate (C)	Lactate (P)	Acetate (P)	Alanine (P)	Isoleucine (C)	Valine (C)	Pyroglutamate (C)
[0 nM]	28.86 ± 14.04	2.67 ± 0.37	61.07 ± 23.8	1.68 ± 0.56	1.05 ± 0.52	0.16 ± 0.39	-0.30 ± 0.47	14.22 ± 1.52
[1 nM]	25.08 ± 12.64	2.44 ± 0.53	45.13 ± 19.75	2.21 ± 1.94	0.81 ± 0.16	0.25 ± 0.21	-0.42 ± 0.08	13.36 ± 1.65
[10 nM]	28.02 ± 11.21	2.83 ± 0.32	46.43 ± 21.21	1.97 ± 0.67	0.81 ± 0.33	0.37 ± 0.34	-0.17 ± 0.22	14.75 ± 1.04
[100 nM]	22.78 ± 9.33	2.47 ± 0.65	54.54 ± 27.81	0.19 ± 0.70	1.01 ± 0.25	0.03 ± 0.26	-0.23 ± 0.35	13.47 ± 1.65

(C)—metabolite consumption; (P)—metabolite production. Subjects were grouped according to body mass index (BMI) and glycemic status (with obesity and euglycemia—Ob+NGT; with obesity and pre-diabetes—Ob+Pre-T2D; with obesity and T2D—Ob+T2D; without obesity—Non-Ob). Data presented as mean ± SEM. * $p < 0.05$ or ** $p < 0.01$ vs GLP-1 [0 nM] of respective experimental group.
